# Utilization of recommended safe-landing strategies during falls in mountain biking

**DOI:** 10.1016/j.heliyon.2024.e40856

**Published:** 2024-12-02

**Authors:** Rickie Ma, Freddie Kim, Sukhman Gosal, Gurvansh Mann, Stephen N. Robinovitch

**Affiliations:** Injury Prevention and Mobility Laboratory, Department of Biomedical Physiology & Kinesiology, Simon Fraser University, Burnaby, British Columbia, Canada

**Keywords:** Accidental falls, Unintentional injuries, Protective responses, Injury prevention, Biomechanics, Bicycling

## Abstract

Falls are common in mountain biking (MTB), and often involve high speeds, large descent heights, and rough landing terrains. However, most falls in MTB do not cause serious injury. This may be due, in part, to protective movements used by MTB riders to avoid injury. Such “safe-landing strategies” are commonly discussed in the MTB community. However, studies have not synthesized or examined the validity of the recommended strategies. Our goal in this study was to determine whether riders utilize recommended safe-landing strategies during real-life falls in MTB. To address this goal, we identified 11 recommended safe-landing strategies through online content analysis and experienced MTB rider surveys. We then analyzed videos of 300 real-life MTB falls using a structured questionnaire to determine whether riders utilized the recommended strategies. The most commonly used strategies were upper limb bracing (58.3 %), elbow flexion at landing (48.0 %), stepping (47.0 %), knee flexion at landing (43.0 %), and bike separation (40.0 %). The least utilized strategies were reach-to-grasp (4.7 %), use of the arms to shield the face (6.3 %) and dismounting from the bike (6.7 %). Moderately utilized strategies included body rolling (26.7 %), neck rotation (26.7 %), and tucking (18.3 %). In 96 % of falls, rider utilized at least 1 recommended landing strategy. On average, riders utilized 3.04 (SD 1.6) recommended landing strategies when falling. Our results indicate that falls in MTB elicit common movement strategies that align with recommended techniques for avoiding injury during falls. Future research should examine the role of exercise in enhancing safe-landing responses and preventing injuries in MTB.

## Highlights

• We examined whether MTB riders use recommended landing strategies during falls.
• We identified 11 recommended “safe-landing” strategies for falls in mountain biking.
• From analysis of real-life falls, riders utilized 3 recommended strategies per fall.
• The most commonly used strategies were upper limb bracing and stepping.
• The least used strategies were dismounting, reach-to-grasp, and shielding the face.

## 1 Introduction

Mountain biking (MTB) is a sport where individuals often pursue paths and features that challenge their ability to maintain balance. Accordingly, falls are common in MTB. Furthermore, falls in MTB often involve high speeds, large descent heights, and landing on rough surface terrain [[1], [2]]. Not surprisingly, up to 85 % of injuries in MTB are caused by falls [[3], [4], [5], [6], [7]].

A more surprising observation, from a biomechanics perspective, is that most falls in MTB do not appear to result in serious injury [[8], [9], [10]]. Among falls in MTB that led to reported injuries, 60 % of injuries were minor cuts and bruises, while only 7 % were bone fractures and 5 % were concussions [5]. We have little understanding of how MTB riders avoid catastrophic injuries when they fall. Helmets and protective padding certainly have an important role in protecting MTB riders. However, additional mechanisms must be acting to mitigate the injury consequences of falls. A better understanding of the factors that separate injurious and non-injurious falls might inform improvements in injury prevention.

The risk for injury during a fall depends, in part, on the use of active responses for orienting the body into a safe-landing configuration, and safely absorbing the energy of the fall. Previous studies have shown that unexpected falls from standing or walking elicit protective movements (or “safe-landing” strategies) such as upper limb bracing to stop the fall before impact occurs to the head, and body rotation to change the direction of the fall [[11], [12], [13], [14], [15], [16], [17]]. However, falling from a bike poses a different situational and environmental context than falls from standing or walking, and may require different approaches for landing safely [9,18]. The question of “how to fall safely” is a common topic in online MTB forums and blogs. However, no study to our knowledge has synthesized the recommendations, and compared the recommended safe-landing strategies to the movement patterns of the body during real-life falls in MTB. Furthermore, the frequency and nature of fall-related injuries in MTB associate with age, sex, and whether MTB is pursued in a recreational versus competitive setting [2,4]. However, no study has examined whether safe-landing strategies in MTB associate with age, sex and setting.

Our goal in this study was to determine whether riders utilize recommended safe-landing strategies during real-life falls in MTB. To accomplish this goal, we addressed three research questions: (1) what strategies are recommended for safe-landing during falls in MTB? (2) how often are the recommended strategies utilized by riders during real-life falls in MTB? and (3) how does the utilization of safe-landing strategies depend on age, sex, and setting (competitive versus recreational MTB)? To answer these questions, we reviewed scientific literature and online content, and conducted surveys with experienced MTB riders, to identify recommended safe-landing strategies. We then analyzed video footage of real-life falls in MTB to determine how often the recommended safe-landing strategies were used during falls experienced by competitive and recreational riders. We discuss the implications of our results on human protective responses that may be relevant to falls in general, and strategies to prevent injuries in MTB through skills training (e.g., instruction on how to land safely during falls), improvements in the design and use of protective clothing, and improvements in trial design (e.g., removal of features that increase risk for injury during falls).

## 2 Methods

### 2.1 Identifying recommended safe-landing strategies

We used three approaches to gather evidence of recommended safe-landing strategies in MTB: online content review, experienced rider surveys, and scientific literature review.

#### 2.1.1 Online content review

We used Google to search online media including articles, blogs, forums, and videos. The search was conducted in “incognito mode” to minimize the likelihood that user data would bias the search results. Furthermore, we logged out of Google accounts, and cleared browser cache and cookies before commencing our search. Two search strings were used: “mountain biking how to crash safely” and “mountain biking how to fall safely”. Additional searches were performed using the native search function on Reddit, specifically targeting the r/MTB subreddit, and on YouTube, with the same search strings. The uniform resource locator (URL) of each search result was saved. Two members of the research team reviewed online content from each search result. Duplicate entries were identified and removed. Documents and videos were included in our analysis if they provided informative or instructional recommendations on safe-landing strategies during falls in MTB. Video transcriptions were obtained from the YouTube transcription feature and subsequently reviewed and edited by the researcher for accuracy. All relevant web content was gathered by NCapture and imported into NVivo14 (Lumivero, 2020). Primary open coding was done with a combination of in-vivo coding (using the exact wording of the source text), and descriptive coding (using phrases that summarized the source text). Axial coding was then done to relate and organize codes. Codes were grouped if they conveyed synonymous strategies, and split to convey different techniques. Finally, larger themes in recommended safe-landing strategies were identified from the codes.

Our online content review yielded 27 sources of information on recommended falling techniques or safe-landing strategies in MTB. These included 8 forum threads, 8 webpages, 6 blog posts, and 5 YouTube video transcripts (see section titled “Online Content References”).

#### 2.1.2 Experienced rider surveys

We conducted surveys, using SurveyMonkey, with experienced MTB riders to gather their opinions on recommended safe-landing strategies for falls in MTB (See Supplementary File 1). We included nine participants (5 males and 4 females), who ranged in age from 26 to 62 years, and had a mean of 16.7 years (SD 12.0; range 2–35) of MTB riding experience. Participants were recruited through notices posted around Simon Fraser University campus and on forums in local online MTB communities. The survey asked the riders to provide brief written answers describing: (1) movement strategies they had personally used to land safely and avoid injury during falls in mountain biking; (2) movement strategies they had personally used to avoid head impact during falls in mountain biking; and (3) recommendations they had read or heard about on movement strategies for landing safely from falls in mountain biking, and the sources. For each question, riders were asked to try to list at least five items (strategies or recommendations), with space provided for additional responses.

On average, participants in the experienced rider survey provided 4.3 (SD 1.1) responses describing strategies they had personally used to land safely and avoid injury during falls in mountain biking, 3.4 (SD 1.1) responses describing strategies they had personally used to avoid head impact (and injury) during falls in mountain biking, and 2.2 (SD 2.0) responses describing recommendations they read or heard about on strategies or techniques for landing safely from a fall in mountain.

#### 2.1.3 Scientific literature review

We searched for peer-reviewed journal articles on PubMed, Web of Science, and SportDiscus, using the keywords “mountain bi∗”, “off-road bi∗”, “fall∗”, “landing”, and “response”. Additional searches were done replacing “bi∗” with “cyc∗”. Each title and abstract were reviewed by two of the authors (RM and FK), and articles were included if they related to safe-landing techniques and strategies during falls in MTB.

We were unable to identify any peer-reviewed journal articles that discussed safe-landing strategies from falls in MTB, or cycling more generally. We identified several peer-reviewed journal articles describing injuries in MTB [2,3,[5], [6], [7],[19], [20], [21]]. However, none of these articles included information on recommended falling techniques or safe-landing strategies from falls in MTB.

#### 2.1.4 Synthesis of recommended safe-landing strategies

Based on information emerging from our online content review and experienced rider surveys, we identified 11 recommended safe-landing strategies that we regarded as observable from watching videos of falls in MTB (Table 1). Strategies that we regarded as unobservable from videos of falls (such as “relax the muscles”) were disregarded.

**Table 1:** Identified safe-landing strategies in the online content review and experienced rider survey.

Recommended safe-landing strategy	Online content review (# of sources; max 27)	Experienced rider survey (# of riders; max 9)	Total # of times mentioned (max 36)
Dismounting	19	7	26
Stepping	11	4	15
Separation from bike	17	7	24
Reach-to-grasp	1	2	3
Rolling	22	7	29
Body tucking	14	7	21
Knee flexion at landing	3	1	4
Upper limb bracing	19^a^	8^b^	27^c^
Elbow flexion at landing	4	4	8
Neck rotation/neck tucking	7	4	11
Shielding the face	3	5	8

NOTES.

a 4 sources encouraged use and 15 sources discouraged use of upper limb bracing.

b 4 experienced riders encouraged use and 4 discouraged use of upper limb bracing.

c 8 total sources encouraged use and 19 total sources discouraged use of upper limb bracing.

### 2.2 Video analysis of real-life safe-landing strategies in MTB

#### 2.2.1 Video footage of real-life falls in MTB

We defined a fall as an imbalance episode that resulted in the rider unintentionally coming to land at a lower level. We defined falls during recreational MTB as those occurring while riders engaged in MTB for leisure or fitness, versus an organized competition. We accessed publicly available video footage of falls in recreational MTB posted to the Pinkbike/crash website. We defined falls in competitive MTB as those occurring on competitive UCI race courses at the regional, national, or international level. We accessed publicly available video footage of falls in competitive MTB from the Vital MTB YouTube channel. These videos are filmed by manned cameras on the sidelines, providing minimally edited and close-up footage of practice runs of Union Cycliste Internationale (UCI) Individual Downhill (DHI) race events.

We only included videos where we could secure information on rider age and sex. For falls in competitive MTB, rider age and sex were determined from online web pages or databases. For falls in recreational riders, rider age and sex were determined by direct messaging with individuals who posted the video on Pinkbike. We also only included videos that provided a clear view of the rider throughout the fall from a third-person perspective, and had a resolution of at least 640 x 480 and a frame rate of at least 15 frames per second. We included 150 falls in competitive riders and 150 falls in recreational MTB that met these criteria. Video footage of each fall was captured using ShareX (version 15.0.0; github.com/ShareX/ShareX), an open-source screen-capturing application. The study was reviewed by the Office of Research Ethics at Simon Fraser University (Study #30000987), and deemed exempt from the requirements for research ethics review, based on Article 2.2 of the Canadian Tri-Council Policy Statement for Ethical Conduct for Research Involving Humans (TCPS2), which states that research does not require REB review when it relies exclusively on information that is in the public domain and the individuals to whom the information refers have no reasonable expectation of privacy.

#### 2.2.2 Rider demographics

For the 300 falls that we analyzed, the mean age of riders at the time of the fall was 24.5 years (SD 9.3) and the median age was 22. For the 150 videos of falls in competitive MTB, the riders had a mean age of 22.7 years (SD = 5.4), and consisted of 133 males (88.7 %) and 17 females (11.3 %). For the 150 videos of falls in recreational MTB, riders had a mean age of 26.3 years (SD = 11.8), and consisted of 143 males (95.3 %) and 7 females (4.7 %).

#### 2.2.3 MTB fall video analysis questionnaire

We developed a questionnaire that probed whether each of the 11 recommended safe-landing strategies were utilized in a given fall (Table 2), for raters to complete while watching the corresponding video. The questionnaire was incorporated into Datavyu (Development release, V1.5.3 for Macintosh), an open-source video coding software package that allows frame-by-frame analysis. Raters were required to answer each question with a response of either “yes” or “no” (“can't tell” responses were not allowed), reflecting the answer they deemed as most correct.

**Table 2:** Inter-rater reliability of recommended safe-landing strategies in MTB.

Recommended safe-landing strategy	Related video analysis question	Total Percent Agreement	Free-marginal Kappa for the question	
Dismounting	Did the rider deliberately dismount (or jump off) the bike between fall initiation and onset of landing?	100 %	1.00	
Stepping	Did the rider deliberately step onto the ground (or an object) after fall initiation?	77.3 %	0.66	
Separation from bike	Was there loss of rider contact with the bike during the fall?	100 %	1.00	
Reach-to-grasp	Did the rider deliberately grasp or contact their hands on an object in the environment after fall initiation?	100 %	1.00	
Rolling	Did the rider roll on the ground when landing from the fall?	70.0 %	0.55	
Knee flexion at landing^a^	Did the rider bend their knee(s) after the feet contacted the ground?	70.0 %	0.55	
Upper limb bracing	Were there deliberate attempts to “arrest the fall” with the upper limbs?	100 %	1.00	
Elbow flexion at landing^a^	Did the rider bend their elbow(s) after contacting their hand(s)?	80.0 %	0.70	
Neck rotation/tucking	Did the rider flex or extend their neck to keep their head off the ground?	80.0 %	0.75	
Shielding the face	Did the rider cover their face or head with their arms, to shield their head from impact?	100 %	1.00	

NOTES.

a Body tucking was defined by a combination of both knee flexion at landing and elbow flexion at landing.

#### 2.2.4 Questionnaire reliability

We examined the inter-rater reliability (and iteratively improved the wording) of each question by comparing responses between two raters (authors RM and SG) who independently reviewed 15 randomly selected fall videos. For each question, reliability was measured based on the total percent agreement between the two raters, and free-marginal kappa [22]. We interpreted kappa values based on the recommendations of Landis and Koch [23]. We observed substantial to near-perfect agreement (k_n_^¥^ ≥ 0.61) for 8 of the 11 questions (Table 2), and moderate agreement for the remaining three questions (0.60 ≥ k_n_^¥^ ≥ 0.41).

#### 2.2.5 Video analysis

Each of the 300 videos were analyzed independently by two raters who were trained by the authors (RM and SG) on how to interpret each question and response category. Raters were able to review, and visualize each video on a frame-by-frame basis, as many times as desired. An instruction manual was provided for better understanding and interpretation of each question. Final answers for a given video were based on the consensus of the two raters. If the two raters differed in their responses to a particular question, a third rater (author RM, FK, or SG) selected the best perceived answer among the two selected.

### 2.3 Data analysis

To address our first research question (concerning the most commonly recommended strategies for safe-landing during falls in MTB), we describe the safe-landing strategies encountered in our online content review, experienced rider surveys, and literature review. We provide quotes to explain the nature of each strategy, and details on the number of sources that recommended each strategy. To address our second research question (on how often the recommended strategies were utilized by riders during real-life falls in MTB), we provide descriptive statistics on the proportion of falls, based on our video analysis, where each recommended safe-landing strategy was utilized. We also report the average number of strategies utilized per fall. To address our third research question (concerning the effects of age, sex and setting on the utilization of safe-landing strategies), we use Pearson chi-squared to test for differences between younger versus older riders, male versus female riders, and competitive versus recreational MTB riders in the percent of falls where each strategy was utilized. Chi-squared is a nonparametric test that does not rely on the data being normally distributed [24]. Significance levels were set *a-priori* at α < 0.05. All statistical analysis was performed using JMP Version 17 (SAS Institute Inc., Cary, NC).

## 3 Results

### 3.1 Recommended safe-landing strategies

Of the 11 recommended safe-landing strategies emerging from our online content review and experienced rider surveys (Table 1), four commence early during the descent stage of a fall (prior to the body contacting the ground). These include “dismounting”, “stepping”, and “separation from the bike”, and “reach-to-grasp”. The remaining seven recommended safe-landing strategies occur during the late descent or landing phase of the fall (“rolling”, “body tucking”, “knee flexion during landing”, “upper limb bracing”, “elbow flexion during landing”, “neck rotation”, and “shielding of the face”). The strategies from most to least commonly recommended were: rolling, dismounting, separation from bike, body tucking, stepping, upper limb fall arrest, neck rotation/neck tucking, elbow flexion at landing, shielding the face, knee flexion at landing, and reach-to-grasp (Table 1).

**Fig. 1:**
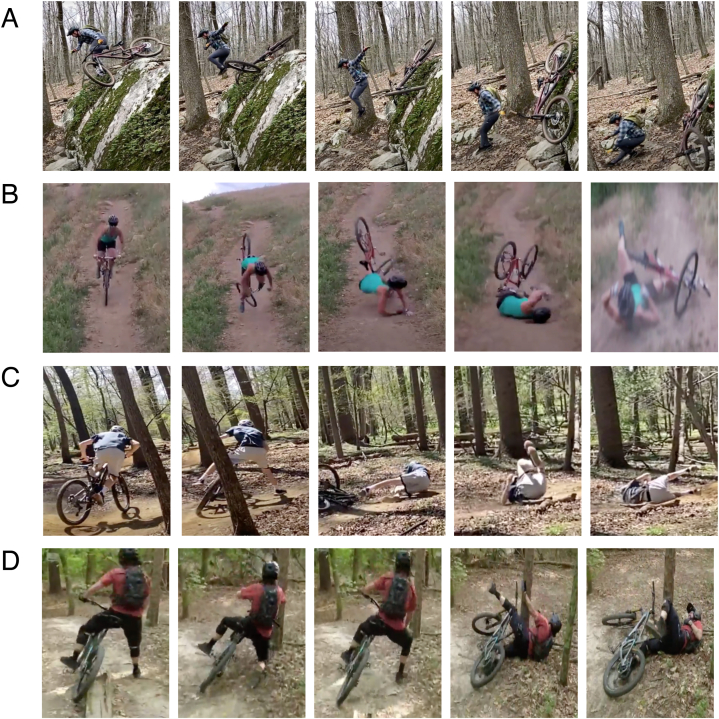
Evidence of riders using recommended “safe-landing” strategies during real-life falls in mountain biking. (A) Fall involving dismounting, knee flexion upon landing, and separation from the bike. (B) Fall involving stepping, neck rotation, upper limb bracing, and elbow flexion upon landing. (C) Fall involving stepping, separation from the bike, and rolling. (D) Fall involving stepping, upper limb bracing, reach-to-grasp, and neck rotation.

***Dismounting*** (or “bailing”) involves hopping off the bike during the descent stage of the fall to land on one's feet (Fig. 1(A)). In the online content review, comments included *“knowing when and how to bail is an underrated skill”* [s14tat, 2021], and *"learning how to quickly jump off your bike can help prevent injuries when faced with a rough trail or obstacle”* [Yardi, 2022]. Comments from the experienced rider survey included “*abandon the bike early! Hop off the side of the bike and land on two feet”* … *“jump off the bike - push off the bike to land on your feet”*, and *“practice jumping off the front of handlebars like hurdles.”*

***Stepping*** (or “dabbing”) is contact of a foot on the ground, or a sequence of steps, to establish a stable standing configuration (Fig. 1(B–D)). A surveyed experienced rider described to *“put a foot down and lay the bike down on the ground”* and *“run out the fall – let go of the bike as it falls and run down the trail/incline/terrain to catch your balance and avoid your body falling”.*

***Separation from the bike*** involves detaching oneself from the bike during descent, often by using the feet and hands to push or throw the bike away from the body, to avoid entanglement or impact with the bicycle (Fig. 1(A)). Comments from the online content review included “*humans are a lot better at rolling on the ground without a bike in between their legs*” [ughsicle22, 2019], and *“your first priority is to distance yourself from the bike by letting go of the bars and unclipping your pedals if necessary”* [Anderson, 2019]. Comments from the experienced rider survey included *“get off the seat (saddle) and separate yourself from the bike”, “jump off/away from bike”* and *“push bike out of the way to prevent entanglement”.*

***Reach-to-grasp*** involves grasping nearby objects such as tree branches or limbs, to halt or slow the body's momentum during the fall (Fig. 1(D)). The surveyed experienced riders mentioned *“reaching out and grabbing hold of a nearby stationary object (i.e., tree)”* and *“grab onto something (tree trunk, etc.) to prevent an impact with the ground”.*

***Rolling*** during landing (as done in a somersault, and commonly used in parkour and judo) spreads the absorption of impact energy over a large surface of successively contacting body parts, and thereby reduces the force applied to a specific region of the body (Fig. 1(C)). Rolling relies on horizontal momentum, which can be generated by early-stage (hand or foot) contacts during landing. In the online content review, the MTB community overwhelmingly advised to perform the rolling technique, especially when catastrophic falls or crashes are unavoidable. One document described that *“the goal is slowing your momentum without forcing yourself to stop. Maintain your tucked position and use your momentum to roll”* [gunnar.o.1, 2022]. In the survey, one rider described *“roll with momentum rather than try to abruptly stop”.*

***Body tucking*** was often mentioned in the context of “tucking and rolling”. Tucking involves placing the elbows and knees close to the torso during landing, to avoid injury to the extremities and allow the torso to absorb the impact energy of the fall. In the online content review, tucking in one's limbs or “balling up” was often recommended. An instructional video described *“you don't want to go hands first straight into that, and you don't want to go legs first straight into it, either. Because all those contact points on the floor have a tendency to just snap under pressure. You just want to tuck and roll 'cause it'd be like a ball; just roll forever. Keep those limbs in”* [Global Mountain Bike Network, 2019]. In the survey, riders described to *“tuck and roll on landing to manage inertia and minimize impact”,* and to *“keeping arms close to the body”.*

***Knee flexion during landing*** is an energy-absorbing technique used in landing from a jump (and in squatting), which involves flexing the knees after landing with one or both feet on the ground (Fig. 1(A)). During knee flexion, the eccentrically-contracting quadriceps muscles act as springs in absorbing energy, slowing downward movement, and reducing the energy that must be absorbed through impact to other body parts [14]. In the online content review, an educational crash prevention video described to avoid the “*joints [from] lock[ing] up, such as if we are coming in straight legged. We can sort of end up in a bit of a squat or a bit of a tumble*” [Pushys Online, 2022]. One experienced rider described to *“land on feet and absorb with knees”.* Another stated to *“avoid landing on a straight leg”.*

***Upper limb bracing*** involves contacting the upper limb on the ground or a nearby object to arrest or brace the fall (Fig. 1(B–D)). In both the online content review and the experienced rider survey, the MTB community held divided views on the benefit of upper limb bracing, surrounding the relative risk of upper limb injury, including fractures, versus the benefit of bracing for preventing impact to the head and face. For example, in the online content review, the consequences of bracing with your upper limbs were described where *“your instincts are to let go of the handlebars and brace yourself for the crash with hands. Despite providing a satisfying level of protection to your chin and face, such an impact usually results in the [impact] of hands, wrists, elbows or collarbones, as the arms are forced to absorb all the kinetic energy in one brief moment”* [Marsal, 2019]. Similarly, many advised to *“not break the fall with your hands or arms”* [ogn3rd, 2018], as “*this mechanism causes injuries so frequently that medical personnel actually have an acronym for it: FOOSH (falling on out-stretched hand)”* [Humphries]. Therefore, others advised to only “*put your [upper limbs] out to absorb the impact”* when doing so in “*a relaxed manner and to avoid tensing up*” [Pushys Online, 2022]. In the survey, one rider recommended *“do not use your arms/hands to absorb your fall”,* while another described sacrificing and *“[brace with] outstretched arms to reduce more serious head injury”.* Among the sources that discussed upper limb bracing, 69 % discouraged its use and 31 % recommended upper limb bracing during MTB falls.

***Elbow flexion during landing*** (Fig. 1(B)) was commonly mentioned in the context of upper limb bracing. Allowing the arm to (partially) collapse during impact reduces the risk for upper limb injury, and facilitates energy absorption in the triceps and pectorals muscles. In the online content review, the MTB community described that “*falling onto [the] forearms rather than straight down onto [the] palms helps prevent broken wrists, collarbones, and shoulders”* [JoshPeck, 2018]*.* In the survey, one rider described *“try not to extend arms in an attempt to brace your landing”*, while another stated to *“avoid landing on a straight arm”.*

***Neck rotation/tucking*** involves rotating the head away from the landing surface (Fig. 1(D)), in a manner that depends on fall direction. One experienced rider recommended *“tucking head away from fall direction”.* Others recommended to “*tuck head toward chest and roll”* and *“tucking in head to avoid it being the first point of impact”.*

***Shielding the face*** involves using the arms and hands to cover and prevent direct impact to the head or face during landing. In the online content review, an educational fall prevention video described that *“you're going to put your hands out and you're definitely going to [brace]. You're going to want to certainly protect your teeth. Get your forearms in front of your face.”* [Pushys Online, 2022]. In the survey, riders recommended *“putting up the arms to protect the face and head from hitting the ground”, “use hands to shield face”,* and *“use arms to protect head”.*

### 3.2 Utilization of recommended safe-landing strategies

***Average number of safe-landing strategies per fall***. On average, riders used 3.0 (SD 1.6) of the recommended safe-landing strategies in a given fall. Riders used 3 or more strategies in 58 % of falls, 5 or more strategies in 20 % of falls, and zero strategies in 4 % of falls. Based on *t*-test, the number of strategies utilized in a fall did not associate with setting (mean (SE) = 3.0 (0.1) for recreational and 3.1 (0.1) for competitive; p = 0.7), age (3.0 (0.1) for younger riders and 3.1 (0.1) for older riders; p = 0.4), but was smaller in female than male riders (2.4 (0.3) versus 3.1(0.1); p = 0.048).

***Utilization of recommended safe-landing strategies during falls.*** Dismounting was observed in 6.7 % of falls (Table 3). Stepping was observed in 47.0 % of falls, with 33.0 % of falls involving a single step and 14.0 % involving multiple steps. Separation from the bike was observed in 40.0 % of falls. Impact with the bike occurred in 18.0 % of falls. Reach-to-grasp was observed in 4.7 % of falls. Rolling was observed in 26.7 % of falls (Table 3). Body tucking was observed in 18.3 % of falls. Knee flexion upon impact was observed in 43.0 % of falls. Upper limb bracing was observed in 58.3 % of falls. Elbow flexion was observed in 48.0 % of all falls, and 70.3 % of falls that involved upper limb bracing. Neck rotation was observed in 26.7 % of falls. Shielding of the face was observed in 6.3 % of falls.

**Table 3:** Percent of falls where recommended safe-landing strategies were utilized, in different settings and rider groups in MTB.

Recommended safe-landing strategy	All falls (n = 300)	Falls in competitive MTB (n = 150)	Falls in recreational MTB (n = 150)	*p*-value	Falls in younger (<23 y/o) riders (n = 154)	Falls in older (≥23 y/o) riders (n = 146)	*p*-value	Falls in males (n = 276)	Falls in females (n = 24)	*p*-value
Dismounting	6.7 %	4.7 %	8.7 %	0.16	4.6 %	8.9 %	0.13	7.2 %	0 %	0.17
Stepping	47.0 %	60.7 %	33.3 %	**<0.0001**	45.5 %	48.6 %	0.58	48.9 %	25.0 %	**0.024**
Separation from bike	40.0 %	35.3 %	44.7 %	0.10	35.7 %	44.5 %	0.12	42.0 %	16.7 %	**0.015**
Reach-to-grasp	4.7 %	4.7 %	4.7 %	1.0	3.9 %	5.5 %	0.52	4.7 %	4.2 %	0.90
Rolling	26.7 %	24.0 %	29.3 %	0.30	24.0 %	29.5 %	0.29	27.5 %	16.7 %	0.25
Body tucking	18.3 %	20.0 %	16.7 %	0.46	20.1 %	16.4 %	0.41	19.6 %	4.2 %	0.062
Knee flexion at landing	43.0 %	52.0 %	34.0 %	**0.002**	44.2 %	41.8 %	0.68	44.2 %	29.2 %	0.15
Upper limb bracing	58.3 %	59.3 %	57.3 %	0.73	61.0 %	55.5 %	0.33	57.2 %	70.8 %	0.20
Elbow flexion at landing	48.0 %	46.0 %	50.0 %	0.49	46.8 %	49.3 %	0.66	47.8 %	50.0 %	0.84
Neck rotation/tucking	26.7 %	20.0 %	33.3 %	**0.009**	28.6 %	24.7 %	0.44	27.5 %	16.7 %	0.25
Shielding the face	6.3 %	3.9 %	9.3 %	**0.033**	4.6 %	8.2 %	0.19	6.2 %	8.3 %	0.67

***Effect of setting, age and sex on the use of safe-landing strategies during falls in MTB.*** There were significant differences between falls in competitive versus recreational MTB settings in the use of four of the 11 safe-landing strategies (Table 3). Stepping and knee flexion were more common during falls in competitive than recreational MTB, while neck rotation and shielding of the face were more common during falls in recreational than competitive MTB. There were no significant differences between younger and older riders in the use of safe-landing strategies (Table 3). There were significant differences between falls in males versus females in the use of two safe-landing strategies (Table 3). Stepping and separation from the bike were more common in males than females.

## 4 Discussion

Previous research has shown that falls from standing or walking trigger safe-landing responses in humans that are tailored to the situational and environmental context of the event [16,25,26]. These responses include stepping and grasping responses for balance recovery [15,27], bracing to arrest the fall with outstretched arms [28], rotation during descent to change the orientation of the fall [26], and knee and hip flexion to reduce the energy of a fall [14]. However, there is a lack of information on safe-landing strategies for falls in MTB, where falls are common, and the consequences of falls can be high.

This study expands our understanding of the mechanics of falls by identifying recommended safe-landing strategies for falls in MTB, and providing evidence on how often the recommended strategies are utilized during real-life MTB falls. We identified 11 recommended safe-landing strategies from online content review and experienced rider surveys. From analyzing video footage of 300 real-life falls in MTB, we found that in most falls, riders used three or more of the recommended safe-landing strategies. Some strategies were used much more often than others. The most commonly utilized strategies were upper limb bracing, elbow flexion at landing, knee flexion at landing, stepping, and bike separation. The least common strategies were reach-to-grasp, face shielding, and dismounting. Utilization of recommended safe-landing strategies associated with MTB setting (competitive versus recreational) and with rider sex, but not with rider age. Stepping was more common during falls in competitive than recreational MTB, while face shielding and neck rotation were more common during falls in recreational than competitive MTB. Stepping and bike separation were more common during falls in males than females.

Several of the recommended strategies align with safe-landing or balance recovery responses that have previously been examined or discussed in the context of falls and fall-related injuries. These include stepping and grasping for balance recovery [15,27], tucking and rolling for maintaining the kinetic energy of the body after landing [[29], [30], [31], [32]], and knee and elbow flexion for energy absorption [16,33], and protecting the head through neck rotation or tucking [34]. However, other recommended strategies were specific to the context of cycling, including dismounting and separation from the bicycle.

The high frequency of upper limb bracing during falls in MTB was surprising, given that sources were more than twice as likely to discourage than encourage its use, citing the danger it posed for upper limb injury. Previous studies found that upper limb bracing increased the risk of upper limb injuries, while decreasing the risk of injury to other body parts [[35], [36], [37]]. In cases where upper limb bracing was encouraged in MTB falls, individuals warned against landing on a straight arm, with the elbows extended. Instead, they recommended landing with the elbows slightly flexed, and continuing to flex the elbow during impact, thereby reducing the total stiffness of the upper limb and the force generated during contact [38,39]. This combination was common during falls in MTB: in 70 % of falls that involved upper limb bracing, we also observed elbow flexion following hand contact.

Our results can inform strategies for preventing injuries through exercise and training, safer trail design, and improvements in equipment. We found that stepping was among the most common safe-landing responses, supporting the value of programs for enhancing rider skills in stepping off the pedals onto the ground [40], and pedal designs that allow for quick removal of the feet [3]. Given the high frequency of upper limb bracing, training should focus on safe bracing through elbow and shoulder flexion during impact [41,42]. It must be acknowledged that complex, technical terrain is an attractive feature to some MTB riders, and unattractive to others. However, in terms of safety, the removal of roots, rocks and logs should facilitate safe-landing responses such as stepping and rolling. Many of the recommended safe-landing responses focused on protecting the head and face against impact and injury, supporting the importance of full face helmets in MTB.

This study has important limitations. First, our dataset of 300 falls may have not fully captured the diversity of rider characteristics, and situational and environmental contexts of falls in MTB. For example, 92 % of the falls we examined were experienced by male riders, and while we found few differences between females and males in utilization of safe-landing strategies, a larger dataset may reveal additional differences. Furthermore, the recreational falls we analyzed may have reflected scenarios that were regarded as entertaining or interesting to the individuals posting the videos, and may not represent the most common types of falls in MTB. However, our analysis focused on the response of the rider to the fall, and none of the falls appeared to be rehearsed or acted out. Second, our results may have been affected by variability between the videos in resolution, frame rate, and the distance and angle of the camera to the fall. However, we observed good inter-rater reliability in the outcomes we report related to the utilization of various falling strategies. Third, we did not examine how the utilization of various strategies depended on fall characteristics such as the direction of the fall, the cause of imbalance, or the activity at the time of the fall. These are important questions for future research. Fourth, we included nine participants in our experienced rider survey, who had a considerable range in age and riding experience. A larger and less heterogeneous sample of MTB riders may have yielded a different range of recommended safe-landing strategies. On the other hand, the similarity in results from our online content review and experienced rider surveys suggests that we achieved saturation in identifying recommended safe-landing strategies for falls in MTB. Fifth, we could only examine falling responses that were observable in the videos, and could not assess “hidden” but potentially important factors such as the thoughts or intentions of the rider during the fall, or the state of muscle excitation at landing. Sixth, we focused on falls in MTB, and our results may not reflect recommended or utilized falling strategies in other contexts, including city bicycling. Finally, we did not assess whether the recommended safe-landing strategies were effective in preventing injuries during falls in MTB. An important next step is to determine whether the recommended safe-falling strategies that we explored are effective in reducing the risk for injury during falls in MTB.

In summary, through online content analysis and experienced rider surveys, we identified 11 recommended safe-landing strategies for falls in MTB. From analysis of video footage of falls in competitive and recreational MTB, we found that each of the recommended safe-landing strategies was utilized to varying degrees, and on average, riders utilized three recommended safe-landing strategies in a given fall. The most utilized strategies were upper limb bracing, elbow flexion at landing, stepping, knee flexion at landing, and bike separation. The least utilized strategies were reach-to-grasp, use of the arms to shield the face, and dismounting from the bike. Moderately utilized strategies included rolling after landing, neck rotation at landing, and tucking. The high prevalence of safe-landing responses may explain why mountain biking falls rarely result in serious injury. Further research is required to compare injury outcomes to utilized landing responses.

## CRediT authorship contribution statement

**Rickie Ma:** Writing – original draft, Validation, Supervision, Methodology, Investigation, Formal analysis, Data curation. **Freddie Kim:** Writing – review & editing, Validation, Methodology, Investigation, Formal analysis, Data curation. **Sukhman Gosal:** Writing – review & editing, Supervision, Methodology, Investigation, Formal analysis, Data curation. **Gurvansh Mann:** Writing – review & editing, Validation, Methodology, Investigation, Formal analysis, Data curation. **Stephen N. Robinovitch:** Writing – review & editing, Writing – original draft, Validation, Supervision, Software, Resources, Project administration, Methodology, Investigation, Funding acquisition, Formal analysis, Data curation, Conceptualization.

## Declaration of competing interest

The authors declare the following financial interests/personal relationships which may be considered as potential competing interests:Stephen Robinovitch reports financial support was provided by 10.13039/501100000038Natural Sciences and Engineering Research Council of Canada. If there are other authors, they declare that they have no known competing financial interests or personal relationships that could have appeared to influence the work reported in this paper.

## ONLINE CONTENT REFERENCES

s14tat. “How important is it to learn to fall/land without getting hurt.” *Reddit*, Jun. 3, 2021, www.reddit.com/r/MTB/comments/nro9dj/how_important_is_it_to_learn_to_fallland_without/.

“Crashing like You Mean It (How Not to Get Hurt).” *Yardi*, Dec. 7, 2022, www.yardimtb.com/blogs/news/crashing-like-you-mean-it-how-not-to-get-hurt.

ughsicle22. “How to learn how to fall?” *Reddit*, Mar. 5, 2019, www.reddit.com/r/MTB/comments/axnrxw/how_to_learn_how_to_fall/.

Anderson. “How to Crash Your EMTB – Tips and Trick to Avoid Bad Injuries.” *E-MOUNTAINBIKE Magazine*, Dec. 3, 2021, www.ebike-mtb.com/en/how-to-crash-your-emtb/.

O, Gunnar. “How to Safely Fall off a Mountain Bike | Curated.Com.” *Curated*, May 12, 2023, www.curated.com/journal/882000/how-to-safely-fall-off-a-mountain-bike.

“How To Crash On Your Mountain Bike | MTB Skills.” *YouTube*, uploaded by Global Mountain Bike Network. 5, Jun. 2019, www.youtube.com/watch?v=NSXbtPG6wy4.

“How to Crash on Your Mountain Bike (And How to Avoid It).” *YouTube*, uploaded by A2B Biking. 9, Aug. 2022, https://www.youtube.com/watch?v=wABzOaG0G1E.

“[R]evolution MTB How to: Crash!” *YouTube*, uploaded by Pushys Online. 28, Apr. 2022, https://www.youtube.com/watch?v=wABzOaG0G1E.

Humphries, David. “How to Fall off of a Mountain Bike without Getting Hurt.” *DIY Mountain Bike*, www.diymountainbike.com/fall-off-mountain-bike/.

Marsal, Adam. “How to Crash as Safely as Possible.” *We Love Cycling Magazine*, We Love Cycling, Oct. 18, 2019, www.welovecycling.com/wide/2019/10/18/how-to-crash-as-safely-as-possible/.

JoshPeck. “More than any helmet, learning how to fall has saved me from severe injury while riding.” *Reddit*, Nov. 23, 2018, https://www.reddit.com/r/MTB/comments/9zrc9w/more_than_any_helmet_learning_how_to_fall_has/
