# Supplementary material for: Utilization of recommended safe-landing strategies during falls in mountain biking
Source: Heliyon. 2024 Dec 2;10(23):e40856. doi: 10.1016/j.heliyon.2024.e40856 (PMC11667004; doi:10.1016/j.heliyon.2024.e40856)
Supplement: Multimedia component 1 [file mmc1.docx]

**SUPPLEMENTARY MATERIAL: Experienced Rider Survey on Safe-Landing Responses in Mountain Biking (MTB)**

**Question 1.** Your own falling experiences in MTB provide clues on the movement strategies that you use to avoid injury during falls. In the text boxes below, please describe the movement strategies you use most often to land safely and avoid injury during falls in MTB. Focus on movements that occur after fall initiation, in the descent and landing phases of the fall. List the strategies from most relevant to least relevant (in your opinion), where relevance is based on how common and protective the strategy is for avoiding injury during falls in MTB.

**Question 2.** Despite the use of helmets, the ability to avoid head impact and injury in falls is an important skill in MTB. In the text boxes below, describe the strategies you use to avoid head impact (and injury) during falls in mountain biking. These can overlap with the strategies described in your answers to Question 1. Focus on movements that occur after fall initiation, in the descent and landing phases of the fall. List the strategies from most relevant to least relevant (in your opinion), where relevance is based on how common and protective the strategy is for avoiding head impact during falls in MTB.

**Question 3.** Perhaps you’ve learned about recommended “safe landing” falling techniques from YouTube videos or internet blogs or forums, or in a mountain biking lesson. This question asks you to describe the recommendations you’ve heard on strategies or techniques for landing safely from a fall in MTB. These may or may not overlap with the strategies that you list under Questions 1 and 2. In the text boxes below, please describe the specific recommendations you’ve heard on strategies or techniques for landing safely from a fall in MTB, and the sources. Focus on movements that occur after fall initiation, in the descent and landing phases of the fall. List the recommendations from most common to least common (in your experience).
